# Supplementary material for: Imputation of 3D genome structure by genetic–epigenetic interaction modeling in mice
Source: eLife. 2024 Apr 26;12:RP88222. doi: 10.7554/eLife.88222 (PMC11052574; doi:10.7554/eLife.88222)
Supplement: Supplementary file 7. [file elife-88222-supp7.zip › Supplemental File 7.html]

STREME Results


Help poup.

[close ]

Motifs discovered by STREME in MEME motif format.

[
close ]

STREME results in XML format.

[
close ]

STREME outputs a tab-separated values (TSV) file ('sequences.tsv') containing one line for
each sequence with a site whose score passes the motif's match threshold for each motif discovered by STREME.
The lines are grouped by motif, and groups are separated by a line
starting with the character "#".
The first line in the file contains the (tab-separated) names of the fields.
The names and meanings of each of the fields are described in the table below.

| field | name | contents |
| --- | --- | --- |
| 1 | motif\_ID | The name of the motif uses the IUPAC codes for nucleotides or proteins. Letters representing multiple nucleotides are used in nucleotide motif positions where several nucleotides are favored. The name of the motif is <index>-<consensus>, where <index> is the rank of the motif according to P-value or Score, and <consensus> is an approximation of the motif by an IUPAC sequence. |
| 2 | motif\_ALT\_ID | The alternate name of the motif is STREME-<index>, where <index> is the rank of the motif according to P-value or Score. |
| 3 | motif\_P-value | The *p*-value of the motif based on applying the appropriate statistical test to the test set sequences. It is not adjusted for the number of motifs reported by STREME. If STREME reports a **single motif**, then the *p*-value is an **accurate estimate** of the statistical significance of the motif as long as the length distributions of the positive and negative sequences are essentially the same. However, if STREME reports more than one motif, the *p*-value does **NOT** completely account for multiple testing, and you should use the *E*-value for assessing whether a motif is truly statistically significant. |
|  | motif\_Score | The Score is the unadjusted *p*-value of the motif based on the appropriate test applied to the training set sequences. Since the Score is not adjusted for multiple tests, it **cannot** be used to determine the statistical significance of the motif. |
| 4 | seq\_ID | The ID of the sequence. |
| 5 | seq\_Score | The seq\_Score of a sequence is its maximum motif match score over all sequence positions. The motif match score of a position in a sequence is computed by summing the appropriate entry from each column of the position-dependent scoring matrix that represents the motif. |
| 5 | seq\_Class | Whether the sequence is a true positive, 'tp', or a false positive, 'fp'. |
| 6 | is\_holdout? | Whether the sequence was in the holdout set, '1', or not, '0'. |

[
close ]

The name of the motif uses the IUPAC codes for nucleotides or proteins.
Letters representing multiple nucleotides are used in nucleotide motif
positions where several nucleotides are favored.
The name of the motif is <index>-<consensus>,
where <index> is the rank of the motif according to P-value or Score,
and <consensus> is an approximation of the motif by an IUPAC sequence.

Read more about the MEME Suite's use of the IUPAC alphabets.

[close ]

The sequence logo of the motif.
The rules for construction logos are given in
the *Description* section of the documentation for the MEME Suite utility
ceqlogo.

[close ]

The sequence logo of the reverse complement motif.
The rules for construction logos are given in
the *Description* section of the documentation for the MEME Suite utility
ceqlogo.

[close ]

Click on the blue symbol below to reveal detailed information about the motif.

[close ]

Click on the blue symbol below to reveal options allowing you
to submit this motif to another MEME Suite motif analysis program, to download this
motif in various text formats, or to download a sequence "logo" of
this motif PNG or EPS format.

##### Supported Programs

Tomtom
:   Tomtom is a tool for searching for similar known motifs.
    [manual]

MAST
:   MAST is a tool for searching biological sequence databases for
    sequences that contain one or more of a group of known motifs.
    [manual]

FIMO
:   FIMO is a tool for searching biological sequence databases for
    sequences that contain one or more known motifs.
    [manual]

GOMo
:   GOMo is a tool for identifying possible roles (Gene Ontology
    terms) for DNA binding motifs.
    [manual]

SpaMo
:   SpaMo is a tool for inferring possible transcription factor
    complexes by finding motifs with enriched spacings.
    [manual]

[close ]

This plot shows the positional distribution of the best match to the motif in the positive training sequences.
Only matches with scores at least the score threshold are considered.
The plot is smoothed with a triangular function whose width is 5% of the maximum positive training sequence length.
The position of the dotted vertical line indicates whether the sequences were
aligned on their left ends, centers, or right ends, respectively.

[
close ]

This histogram shows the distribution of the **number** of matches to the motif in the positive training sequences with at least one match.
Only matches with scores at least the score threshold are considered.

[
close ]

The number of positive sequences matching the motif (percentage).

[close ]

The number of training set positive sequences matching the motif / the number of training set positive sequences.

Note these counts are made after erasing sites that match previously
found motifs.

[close ]

The number of training set positive sequences matching the motif.

Note these counts are made after erasing sites that match previously
found motifs.

[close ]

The number of training set negative sequences matching the motif / the number of training set negative sequences.

Note these counts are made after erasing sites that match previously
found motifs.

[close ]

The number test set positive sequences matching the motif / the number of test set positive sequences.

Note these counts are made after erasing sites that match previously
found motifs.

[close ]

The number of test set positive sequences matching the motif.

Note these counts are made after erasing sites that match previously
found motifs.

[close ]

The number of test set negative sequences matching the motif / the number of test set negative sequences.

Note these counts are made after erasing sites that match previously
found motifs.

[close ]

The mean distance from the center of the best match to the sequence center,
averaged over all training set sequences with a match.

[close ]

The mean distance from the center of the best match to the sequence center,
averaged over all test set sequences with a match.

[close ]

The Score is the unadjusted *p*-value of the motif based on the appropriate test
applied to the training set sequences.
Since the Score is not adjusted for multiple tests, it **cannot**
be used to determine the statistical significance of the motif.

For determining if a motif is statistically significant, you should use the
value in the E-value column. If there is no E-value column, that
means that either the positive or negative hold-out set would have been too small
(fewer than 5 sequences).
For very small sequence sets, it is not practical for STREME to compute an accurate *E*-value.
In that case, you can determine if your motif is significant by running STREME twenty or more
times on shuffled versions of your positive dataset,
and seeing if the Score is always **larger** than the Score using the original sequences.
You can make shuffled sequence datasets using the MEME Suite command-line utility
fasta-shuffle-letters) if you
have installed the MEME Suite on your own computer.

The statistical test used in computing the Score is either the Fisher Exact Test,
the Binomial Test, or the Cumulative Bates distribution. (See Inputs and Settings
for the particular test being used.) The Fisher Exact Test and the Binomial Test both
estimate the enrichment of the motif in the positive sequences compared to the
the negative sequences.
(The Binomial Test is used when the positive and negative sequences have different average lengths.)
The Cumulative Bates distribution measures the tendency
of motif to be near the center of the input sequences.

[close ]

The *p*-value of the motif based on applying the appropriate statistical test
to the test set sequences. It is not adjusted for the number of motifs reported by STREME.

If STREME reports a **single motif**, then
the *p*-value is an **accurate estimate** of
the statistical significance of the motif as long as the length
distributions of the positive and negative sequences are essentially the same.
However, if STREME reports more than one motif, the *p*-value does **NOT**
completely account for multiple testing, and you should use the *E*-value
for assessing whether a motif is truly statistically significant.

The statistical test used in computing the *p*-value is either the Fisher Exact Test,
the Binomial Test, or the Cumulative Bates distribution. (See Inputs and Settings
at the bottom of this document for the particular test being used.)
The Fisher Exact Test and the Binomial Test both
measure the enrichment of the motif in the positive test sequences compared to the
the negative test sequences.
(The Binomial Test is used when the positive and negative sequences have different average lengths.)
The Cumulative Bates distribution measures the tendency
of motif to be near the center of the sequences.

[close ]

The *E*-value is an **accurate estimate** of
the statistical significance of the motif as long as the length
distributions of the positive and negative sequences are essentially the same.
The *E*-value is the *p*-value multiplied by the number of motifs reported by STREME.
It is an estimate of the number of motifs that would be found with enrichment
as high as this motif in shuffled versions of your positive sequences.

[close ]

The score threshold for determining if a potential site is a match
to the motif. The same threshold is applied when determining matches in
the training and test sequences. The threshold is in bits.

The match score of a position in a sequence is determined by converting
the motif to a base-2 log-odds matrix using the formula log2(prob[a][i]/background[a]).
Here, prob[a][i] is the probability of the letter 'a' at position 'i' of the motif,
and background[a] is the probability of the letter 'a' according to the background.

[close ]

The names of the files containing the positive (primary)
and negative (control) sequences input to STREME.

If you did not provide a file containing the negative (e.g., control)
sequences, STREME created them using N-order shuffling.
0-order shuffling preserves 1-mer frequencies (i.e., the letter frequencies),
1-order shuffling preserves 2-mer frequencies, etc.

[close ]

The name of the alphabet of the sequences.

[close ]

The number of sequences.

[close ]

The total length of the sequences.

[close ]

The name of the alphabet symbol.

[close ]

The frequency of the alphabet symbol in the negative sequences.

[close ]

The frequency of the alphabet symbol as defined by the background model.

[
close ]

#### Details

| Train Positives | Train Positives | Train Negatives | Train DTC | Score | Test Positives | Test Positives | Test Negatives | Test DTC | P-value | Match Threshold |
| --- | --- | --- | --- | --- | --- | --- | --- | --- | --- | --- |
| /  () | /  () |  |  |  | /  () | /  () |  |  |  |  |

x

## Submit or Download

⇧⬆

⇩⬇

Submit MotifDownload MotifDownload Logo

#### Submit to program

|  |  |  |
| --- | --- | --- |
|  | Tomtom | Find similar motifs in published libraries or a library you supply. |
|  | FIMO | Find motif occurrences in sequence data. |
|  | MAST | Rank sequences by affinity to groups of motifs. |
|  | GOMo | Identify possible roles (Gene Ontology terms) for motifs. |
|  | SpaMo | Find other motifs that are enriched at specific close spacings which might imply the existence of a complex. |

Format:

Count Matrix
Probability Matrix
Minimal MEME

|  |  |
| --- | --- |
| Format: | PNG (for web) EPS (for publication) |
| Orientation: | Normal Reverse Complement |
| Small Sample Correction: | Off On |
| Width: | cm |
| Height: | cm |

# STREME

## Sensitive, Thorough, Rapid, Enriched Motif Elicitation

For further information on how to interpret these results please access
https://meme-suite.org/meme/doc/streme.html.   
To get a copy of the MEME software please access
https://meme-suite.org.

If you use STREME in your research, please cite the following paper:  

Timothy L. Bailey,
"STREME: accurate and versatile sequence motif discovery",
*Bioinformatics*, Mar. 24, 2021.
[full text]

Discovered Motifs
  |  
Inputs & Settings
  |  
Program Information
  |  
Motifs in MEME Text Format 
  |  
Matching Sequences  
  |  
Results in XML Format


# Javascript is required to view these results!

# Your browser does not support canvas!


## Description

neg50 vs shuffled


## Discovered Motifs

Next Top

| Motif | Logo | RC Logo | P-value | E-value | Sites | More | Submit/Download | Positional Distribution | Matches per Sequence |
| --- | --- | --- | --- | --- | --- | --- | --- | --- | --- |
| 1-CCCCACCCCCACC |  |  | 1.4e-022 | 7.1e-021 | 1338 (28.1%) | ↧↥ | ⇢ |  |  |
| Details  | Train Positives | Train Positives | Train Negatives | Train DTC | Score | Test Positives | Test Positives | Test Negatives | Test DTC | P-value | Match Threshold | | --- | --- | --- | --- | --- | --- | --- | --- | --- | --- | --- | | 1200 / 4266 (28.1%) | 185 / 4286 (4.3%) | 1200 | -1 | 1.3e-182 | 138 / 492 (28.0%) | 21 / 492 (4.3%) | 138 | -1 | 1.4e-022 | 12.3423 | | | | | | | |
| Motif | Logo | RC Logo | P-value | E-value | Sites | More | Submit/Download | Positional Distribution | Matches per Sequence |
| 2-ACACACACACACACA |  |  | 1.7e-014 | 8.6e-013 | 532 (11.2%) | ↧↥ | ⇢ |  |  |
| Details  | Train Positives | Train Positives | Train Negatives | Train DTC | Score | Test Positives | Test Positives | Test Negatives | Test DTC | P-value | Match Threshold | | --- | --- | --- | --- | --- | --- | --- | --- | --- | --- | --- | | 474 / 4266 (11.1%) | 41 / 4286 (1.0%) | 474 | -1 | 9.2e-095 | 58 / 492 (11.8%) | 3 / 492 (0.6%) | 58 | -1 | 1.7e-014 | 14.3252 | | | | | | | |
| Motif | Logo | RC Logo | P-value | E-value | Sites | More | Submit/Download | Positional Distribution | Matches per Sequence |
| 3-RCCASMAGAGGGCR |  |  | 2.0e-014 | 1.0e-012 | 607 (12.8%) | ↧↥ | ⇢ |  |  |
| Details  | Train Positives | Train Positives | Train Negatives | Train DTC | Score | Test Positives | Test Positives | Test Negatives | Test DTC | P-value | Match Threshold | | --- | --- | --- | --- | --- | --- | --- | --- | --- | --- | --- | | 546 / 4266 (12.8%) | 52 / 4286 (1.2%) | 546 | -1 | 3.4e-105 | 61 / 492 (12.4%) | 4 / 492 (0.8%) | 61 | -1 | 2.0e-014 | 13.7353 | | | | | | | |
| Motif | Logo | RC Logo | P-value | E-value | Sites | More | Submit/Download | Positional Distribution | Matches per Sequence |
| 4-AAAAAAAAAAAAAAA |  |  | 2.0e-014 | 1.0e-012 | 1102 (23.2%) | ↧↥ | ⇢ |  |  |
| Details  | Train Positives | Train Positives | Train Negatives | Train DTC | Score | Test Positives | Test Positives | Test Negatives | Test DTC | P-value | Match Threshold | | --- | --- | --- | --- | --- | --- | --- | --- | --- | --- | --- | | 989 / 4266 (23.2%) | 127 / 4286 (3.0%) | 989 | -1 | 2.9e-166 | 113 / 492 (23.0%) | 26 / 492 (5.3%) | 113 | -1 | 2.0e-014 | 12.7113 | | | | | | | |
| Motif | Logo | RC Logo | P-value | E-value | Sites | More | Submit/Download | Positional Distribution | Matches per Sequence |
| 5-CCTGCCTCTGCCTC |  |  | 3.0e-012 | 1.5e-010 | 546 (11.5%) | ↧↥ | ⇢ |  |  |
| Details  | Train Positives | Train Positives | Train Negatives | Train DTC | Score | Test Positives | Test Positives | Test Negatives | Test DTC | P-value | Match Threshold | | --- | --- | --- | --- | --- | --- | --- | --- | --- | --- | --- | | 493 / 4266 (11.6%) | 43 / 4286 (1.0%) | 493 | -1 | 3.3e-098 | 53 / 492 (10.8%) | 4 / 492 (0.8%) | 53 | -1 | 3.0e-012 | 14.2269 | | | | | | | |
| Motif | Logo | RC Logo | P-value | E-value | Sites | More | Submit/Download | Positional Distribution | Matches per Sequence |
| 6-AGAGAGAGAGAGAGA |  |  | 3.7e-012 | 1.9e-010 | 323 (6.8%) | ↧↥ | ⇢ |  |  |
| Details  | Train Positives | Train Positives | Train Negatives | Train DTC | Score | Test Positives | Test Positives | Test Negatives | Test DTC | P-value | Match Threshold | | --- | --- | --- | --- | --- | --- | --- | --- | --- | --- | --- | | 285 / 4266 (6.7%) | 47 / 4286 (1.1%) | 285 | -1 | 5.4e-043 | 38 / 492 (7.7%) | 0 / 492 (0.0%) | 38 | -1 | 3.7e-012 | 14.5767 | | | | | | | |
| Motif | Logo | RC Logo | P-value | E-value | Sites | More | Submit/Download | Positional Distribution | Matches per Sequence |
| 7-GAGTTCCAGGMCAGC |  |  | 1.2e-010 | 6.3e-009 | 491 (10.3%) | ↧↥ | ⇢ |  |  |
| Details  | Train Positives | Train Positives | Train Negatives | Train DTC | Score | Test Positives | Test Positives | Test Negatives | Test DTC | P-value | Match Threshold | | --- | --- | --- | --- | --- | --- | --- | --- | --- | --- | --- | | 444 / 4266 (10.4%) | 6 / 4286 (0.1%) | 444 | -1 | 4.0e-123 | 47 / 492 (9.6%) | 4 / 492 (0.8%) | 47 | -1 | 1.2e-010 | 14.402 | | | | | | | |
| Motif | Logo | RC Logo | P-value | E-value | Sites | More | Submit/Download | Positional Distribution | Matches per Sequence |
| 8-TGAGCCATCTCTCCA |  |  | 3.0e-008 | 1.6e-006 | 235 (4.9%) | ↧↥ | ⇢ |  |  |
| Details  | Train Positives | Train Positives | Train Negatives | Train DTC | Score | Test Positives | Test Positives | Test Negatives | Test DTC | P-value | Match Threshold | | --- | --- | --- | --- | --- | --- | --- | --- | --- | --- | --- | | 210 / 4266 (4.9%) | 3 / 4286 (0.1%) | 210 | -1 | 1.2e-058 | 25 / 492 (5.1%) | 0 / 492 (0.0%) | 25 | -1 | 3.0e-008 | 14.8894 | | | | | | | |
| Motif | Logo | RC Logo | P-value | E-value | Sites | More | Submit/Download | Positional Distribution | Matches per Sequence |
| 9-AGACAGGGTTTCTCT |  |  | 1.2e-007 | 6.2e-006 | 230 (4.8%) | ↧↥ | ⇢ |  |  |
| Details  | Train Positives | Train Positives | Train Negatives | Train DTC | Score | Test Positives | Test Positives | Test Negatives | Test DTC | P-value | Match Threshold | | --- | --- | --- | --- | --- | --- | --- | --- | --- | --- | --- | | 207 / 4266 (4.9%) | 1 / 4286 (0.0%) | 207 | -1 | 5.1e-061 | 23 / 492 (4.7%) | 0 / 492 (0.0%) | 23 | -1 | 1.2e-007 | 16.2396 | | | | | | | |
| Motif | Logo | RC Logo | P-value | E-value | Sites | More | Submit/Download | Positional Distribution | Matches per Sequence |
| 10-CCTTTAATCCCAGCA |  |  | 1.2e-007 | 6.4e-006 | 383 (8.0%) | ↧↥ | ⇢ |  |  |
| Details  | Train Positives | Train Positives | Train Negatives | Train DTC | Score | Test Positives | Test Positives | Test Negatives | Test DTC | P-value | Match Threshold | | --- | --- | --- | --- | --- | --- | --- | --- | --- | --- | --- | | 353 / 4266 (8.3%) | 6 / 4286 (0.1%) | 353 | -1 | 2.5e-096 | 30 / 492 (6.1%) | 2 / 492 (0.4%) | 30 | -1 | 1.2e-007 | 14.2737 | | | | | | | |
| Motif | Logo | RC Logo | P-value | E-value | Sites | More | Submit/Download | Positional Distribution | Matches per Sequence |
| 11-CCCACCCCACCCC |  |  | 1.9e-007 | 1.0e-005 | 681 (14.3%) | ↧↥ | ⇢ |  |  |
| Details  | Train Positives | Train Positives | Train Negatives | Train DTC | Score | Test Positives | Test Positives | Test Negatives | Test DTC | P-value | Match Threshold | | --- | --- | --- | --- | --- | --- | --- | --- | --- | --- | --- | | 621 / 4266 (14.6%) | 183 / 4286 (4.3%) | 621 | -1 | 8.8e-057 | 60 / 492 (12.2%) | 16 / 492 (3.3%) | 60 | -1 | 1.9e-007 | 12.0644 | | | | | | | |
| Motif | Logo | RC Logo | P-value | E-value | Sites | More | Submit/Download | Positional Distribution | Matches per Sequence |
| 12-GGAGGGAGGGAGGGA |  |  | 3.7e-007 | 1.9e-005 | 478 (10.0%) | ↧↥ | ⇢ |  |  |
| Details  | Train Positives | Train Positives | Train Negatives | Train DTC | Score | Test Positives | Test Positives | Test Negatives | Test DTC | P-value | Match Threshold | | --- | --- | --- | --- | --- | --- | --- | --- | --- | --- | --- | | 433 / 4266 (10.2%) | 101 / 4286 (2.4%) | 433 | -1 | 3.1e-050 | 45 / 492 (9.1%) | 9 / 492 (1.8%) | 45 | -1 | 3.7e-007 | 13.2674 | | | | | | | |
| Motif | Logo | RC Logo | P-value | E-value | Sites | More | Submit/Download | Positional Distribution | Matches per Sequence |
| 13-GCCCCGCCCC |  |  | 4.9e-007 | 2.5e-005 | 780 (16.4%) | ↧↥ | ⇢ |  |  |
| Details  | Train Positives | Train Positives | Train Negatives | Train DTC | Score | Test Positives | Test Positives | Test Negatives | Test DTC | P-value | Match Threshold | | --- | --- | --- | --- | --- | --- | --- | --- | --- | --- | --- | | 703 / 4266 (16.5%) | 201 / 4286 (4.7%) | 703 | -1 | 3.6e-066 | 77 / 492 (15.7%) | 27 / 492 (5.5%) | 77 | -1 | 4.9e-007 | 12.0962 | | | | | | | |
| Motif | Logo | RC Logo | P-value | E-value | Sites | More | Submit/Download | Positional Distribution | Matches per Sequence |
| 14-CRTGGTGGCKCACR |  |  | 1.3e-006 | 6.7e-005 | 353 (7.4%) | ↧↥ | ⇢ |  |  |
| Details  | Train Positives | Train Positives | Train Negatives | Train DTC | Score | Test Positives | Test Positives | Test Negatives | Test DTC | P-value | Match Threshold | | --- | --- | --- | --- | --- | --- | --- | --- | --- | --- | --- | | 324 / 4266 (7.6%) | 24 / 4286 (0.6%) | 324 | -1 | 1.4e-068 | 29 / 492 (5.9%) | 3 / 492 (0.6%) | 29 | -1 | 1.3e-006 | 13.8027 | | | | | | | |
| Motif | Logo | RC Logo | P-value | E-value | Sites | More | Submit/Download | Positional Distribution | Matches per Sequence |
| 15-CTGGTCTACARAGT |  |  | 2.8e-006 | 1.5e-004 | 291 (6.1%) | ↧↥ | ⇢ |  |  |
| Details  | Train Positives | Train Positives | Train Negatives | Train DTC | Score | Test Positives | Test Positives | Test Negatives | Test DTC | P-value | Match Threshold | | --- | --- | --- | --- | --- | --- | --- | --- | --- | --- | --- | | 266 / 4266 (6.2%) | 2 / 4286 (0.0%) | 266 | -1 | 7.7e-077 | 25 / 492 (5.1%) | 2 / 492 (0.4%) | 25 | -1 | 2.8e-006 | 15.1425 | | | | | | | |
| Motif | Logo | RC Logo | P-value | E-value | Sites | More | Submit/Download | Positional Distribution | Matches per Sequence |
| 16-CGCCGCCGCCGCCG |  |  | 7.6e-006 | 4.0e-004 | 301 (6.3%) | ↧↥ | ⇢ |  |  |
| Details  | Train Positives | Train Positives | Train Negatives | Train DTC | Score | Test Positives | Test Positives | Test Negatives | Test DTC | P-value | Match Threshold | | --- | --- | --- | --- | --- | --- | --- | --- | --- | --- | --- | | 275 / 4266 (6.4%) | 18 / 4286 (0.4%) | 275 | -1 | 1.6e-060 | 26 / 492 (5.3%) | 3 / 492 (0.6%) | 26 | -1 | 7.6e-006 | 16.0123 | | | | | | | |
| Motif | Logo | RC Logo | P-value | E-value | Sites | More | Submit/Download | Positional Distribution | Matches per Sequence |
| 17-TGCTGGGAWTTGAAC |  |  | 1.3e-005 | 6.6e-004 | 363 (7.6%) | ↧↥ | ⇢ |  |  |
| Details  | Train Positives | Train Positives | Train Negatives | Train DTC | Score | Test Positives | Test Positives | Test Negatives | Test DTC | P-value | Match Threshold | | --- | --- | --- | --- | --- | --- | --- | --- | --- | --- | --- | | 329 / 4266 (7.7%) | 34 / 4286 (0.8%) | 329 | -1 | 4.5e-062 | 34 / 492 (6.9%) | 7 / 492 (1.4%) | 34 | -1 | 1.3e-005 | 13.5008 | | | | | | | |
| Motif | Logo | RC Logo | P-value | E-value | Sites | More | Submit/Download | Positional Distribution | Matches per Sequence |
| 18-AGTGCTCTTAACCRC |  |  | 3.1e-005 | 1.6e-003 | 156 (3.3%) | ↧↥ | ⇢ |  |  |
| Details  | Train Positives | Train Positives | Train Negatives | Train DTC | Score | Test Positives | Test Positives | Test Negatives | Test DTC | P-value | Match Threshold | | --- | --- | --- | --- | --- | --- | --- | --- | --- | --- | --- | | 141 / 4266 (3.3%) | 1 / 4286 (0.0%) | 141 | -1 | 2.6e-041 | 15 / 492 (3.0%) | 0 / 492 (0.0%) | 15 | -1 | 3.1e-005 | 16.6592 | | | | | | | |
| Motif | Logo | RC Logo | P-value | E-value | Sites | More | Submit/Download | Positional Distribution | Matches per Sequence |
| 19-AAATAAATAT |  |  | 6.1e-005 | 3.2e-003 | 919 (19.3%) | ↧↥ | ⇢ |  |  |
| Details  | Train Positives | Train Positives | Train Negatives | Train DTC | Score | Test Positives | Test Positives | Test Negatives | Test DTC | P-value | Match Threshold | | --- | --- | --- | --- | --- | --- | --- | --- | --- | --- | --- | | 819 / 4266 (19.2%) | 359 / 4286 (8.4%) | 819 | -1 | 3.9e-042 | 100 / 492 (20.3%) | 52 / 492 (10.6%) | 100 | -1 | 6.1e-005 | 11.2015 | | | | | | | |
| Motif | Logo | RC Logo | P-value | E-value | Sites | More | Submit/Download | Positional Distribution | Matches per Sequence |
| 20-CAGGGCTAYAC |  |  | 1.4e-004 | 7.2e-003 | 203 (4.3%) | ↧↥ | ⇢ |  |  |
| Details  | Train Positives | Train Positives | Train Negatives | Train DTC | Score | Test Positives | Test Positives | Test Negatives | Test DTC | P-value | Match Threshold | | --- | --- | --- | --- | --- | --- | --- | --- | --- | --- | --- | | 187 / 4266 (4.4%) | 15 / 4286 (0.3%) | 187 | -1 | 2.9e-039 | 16 / 492 (3.3%) | 1 / 492 (0.2%) | 16 | -1 | 1.4e-004 | 15.1092 | | | | | | | |
| Motif | Logo | RC Logo | P-value | E-value | Sites | More | Submit/Download | Positional Distribution | Matches per Sequence |
| 21-CCCCTCCCC |  |  | 1.7e-004 | 8.8e-003 | 433 (9.1%) | ↧↥ | ⇢ |  |  |
| Details  | Train Positives | Train Positives | Train Negatives | Train DTC | Score | Test Positives | Test Positives | Test Negatives | Test DTC | P-value | Match Threshold | | --- | --- | --- | --- | --- | --- | --- | --- | --- | --- | --- | | 387 / 4266 (9.1%) | 203 / 4286 (4.7%) | 387 | -1 | 1.5e-014 | 46 / 492 (9.3%) | 17 / 492 (3.5%) | 46 | -1 | 1.7e-004 | 14.2237 | | | | | | | |
| Motif | Logo | RC Logo | P-value | E-value | Sites | More | Submit/Download | Positional Distribution | Matches per Sequence |
| 22-AGGAAGCAG |  |  | 2.4e-004 | 1.3e-002 | 1332 (28.0%) | ↧↥ | ⇢ |  |  |
| Details  | Train Positives | Train Positives | Train Negatives | Train DTC | Score | Test Positives | Test Positives | Test Negatives | Test DTC | P-value | Match Threshold | | --- | --- | --- | --- | --- | --- | --- | --- | --- | --- | --- | | 1203 / 4266 (28.2%) | 815 / 4286 (19.0%) | 1203 | -1 | 2.8e-018 | 129 / 492 (26.2%) | 78 / 492 (15.9%) | 129 | -1 | 2.4e-004 | 10.1601 | | | | | | | |
| Motif | Logo | RC Logo | P-value | E-value | Sites | More | Submit/Download | Positional Distribution | Matches per Sequence |
| 23-AAAACAAA |  |  | 3.2e-004 | 1.6e-002 | 484 (10.2%) | ↧↥ | ⇢ |  |  |
| Details  | Train Positives | Train Positives | Train Negatives | Train DTC | Score | Test Positives | Test Positives | Test Negatives | Test DTC | P-value | Match Threshold | | --- | --- | --- | --- | --- | --- | --- | --- | --- | --- | --- | | 426 / 4266 (10.0%) | 233 / 4286 (5.4%) | 426 | -1 | 2.5e-014 | 58 / 492 (11.8%) | 26 / 492 (5.3%) | 58 | -1 | 3.2e-004 | 12.989 | | | | | | | |
| Motif | Logo | RC Logo | P-value | E-value | Sites | More | Submit/Download | Positional Distribution | Matches per Sequence |
| 24-ATGCAAATD |  |  | 4.6e-004 | 2.4e-002 | 533 (11.2%) | ↧↥ | ⇢ |  |  |
| Details  | Train Positives | Train Positives | Train Negatives | Train DTC | Score | Test Positives | Test Positives | Test Negatives | Test DTC | P-value | Match Threshold | | --- | --- | --- | --- | --- | --- | --- | --- | --- | --- | --- | | 482 / 4266 (11.3%) | 197 / 4286 (4.6%) | 482 | -1 | 9.1e-029 | 51 / 492 (10.4%) | 22 / 492 (4.5%) | 51 | -1 | 4.6e-004 | 12.6862 | | | | | | | |
| Motif | Logo | RC Logo | P-value | E-value | Sites | More | Submit/Download | Positional Distribution | Matches per Sequence |
| 25-CCTYTGGAAGAGCAG |  |  | 4.9e-004 | 2.5e-002 | 166 (3.5%) | ↧↥ | ⇢ |  |  |
| Details  | Train Positives | Train Positives | Train Negatives | Train DTC | Score | Test Positives | Test Positives | Test Negatives | Test DTC | P-value | Match Threshold | | --- | --- | --- | --- | --- | --- | --- | --- | --- | --- | --- | | 155 / 4266 (3.6%) | 8 / 4286 (0.2%) | 155 | -1 | 9.4e-037 | 11 / 492 (2.2%) | 0 / 492 (0.0%) | 11 | -1 | 4.9e-004 | 15.134 | | | | | | | |
| Motif | Logo | RC Logo | P-value | E-value | Sites | More | Submit/Download | Positional Distribution | Matches per Sequence |
| 26-CCCTTCCCC |  |  | 8.9e-004 | 4.6e-002 | 293 (6.2%) | ↧↥ | ⇢ |  |  |
| Details  | Train Positives | Train Positives | Train Negatives | Train DTC | Score | Test Positives | Test Positives | Test Negatives | Test DTC | P-value | Match Threshold | | --- | --- | --- | --- | --- | --- | --- | --- | --- | --- | --- | | 256 / 4266 (6.0%) | 130 / 4286 (3.0%) | 256 | -1 | 6.9e-011 | 37 / 492 (7.5%) | 14 / 492 (2.8%) | 37 | -1 | 8.9e-004 | 14.8268 | | | | | | | |
| Motif | Logo | RC Logo | P-value | E-value | Sites | More | Submit/Download | Positional Distribution | Matches per Sequence |
| 27-CTCCTCCTCCTCCYY |  |  | 1.8e-003 | 9.2e-002 | 880 (18.5%) | ↧↥ | ⇢ |  |  |
| Details  | Train Positives | Train Positives | Train Negatives | Train DTC | Score | Test Positives | Test Positives | Test Negatives | Test DTC | P-value | Match Threshold | | --- | --- | --- | --- | --- | --- | --- | --- | --- | --- | --- | | 811 / 4266 (19.0%) | 359 / 4286 (8.4%) | 811 | -1 | 5.5e-041 | 69 / 492 (14.0%) | 38 / 492 (7.7%) | 69 | -1 | 1.8e-003 | 11.3199 | | | | | | | |
| Motif | Logo | RC Logo | P-value | E-value | Sites | More | Submit/Download | Positional Distribution | Matches per Sequence |
| 28-CTCCACCC |  |  | 2.1e-003 | 1.1e-001 | 583 (12.3%) | ↧↥ | ⇢ |  |  |
| Details  | Train Positives | Train Positives | Train Negatives | Train DTC | Score | Test Positives | Test Positives | Test Negatives | Test DTC | P-value | Match Threshold | | --- | --- | --- | --- | --- | --- | --- | --- | --- | --- | --- | | 516 / 4266 (12.1%) | 341 / 4286 (8.0%) | 516 | -1 | 1.2e-009 | 67 / 492 (13.6%) | 37 / 492 (7.5%) | 67 | -1 | 2.1e-003 | 13.393 | | | | | | | |
| Motif | Logo | RC Logo | P-value | E-value | Sites | More | Submit/Download | Positional Distribution | Matches per Sequence |
| 29-TTAAAAAWAAA |  |  | 2.7e-003 | 1.4e-001 | 730 (15.3%) | ↧↥ | ⇢ |  |  |
| Details  | Train Positives | Train Positives | Train Negatives | Train DTC | Score | Test Positives | Test Positives | Test Negatives | Test DTC | P-value | Match Threshold | | --- | --- | --- | --- | --- | --- | --- | --- | --- | --- | --- | | 676 / 4266 (15.8%) | 280 / 4286 (6.5%) | 676 | -1 | 9.4e-039 | 54 / 492 (11.0%) | 28 / 492 (5.7%) | 54 | -1 | 2.7e-003 | 12.1364 | | | | | | | |
| Motif | Logo | RC Logo | P-value | E-value | Sites | More | Submit/Download | Positional Distribution | Matches per Sequence |
| 30-CGCGCGCGCGCG |  |  | 3.2e-003 | 1.7e-001 | 301 (6.3%) | ↧↥ | ⇢ |  |  |
| Details  | Train Positives | Train Positives | Train Negatives | Train DTC | Score | Test Positives | Test Positives | Test Negatives | Test DTC | P-value | Match Threshold | | --- | --- | --- | --- | --- | --- | --- | --- | --- | --- | --- | | 272 / 4266 (6.4%) | 91 / 4286 (2.1%) | 272 | -1 | 2.3e-022 | 29 / 492 (5.9%) | 11 / 492 (2.2%) | 29 | -1 | 3.2e-003 | 12.966 | | | | | | | |
| Motif | Logo | RC Logo | P-value | E-value | Sites | More | Submit/Download | Positional Distribution | Matches per Sequence |
| 31-CCCGCCCCC |  |  | 3.8e-003 | 2.0e-001 | 379 (8.0%) | ↧↥ | ⇢ |  |  |
| Details  | Train Positives | Train Positives | Train Negatives | Train DTC | Score | Test Positives | Test Positives | Test Negatives | Test DTC | P-value | Match Threshold | | --- | --- | --- | --- | --- | --- | --- | --- | --- | --- | --- | | 340 / 4266 (8.0%) | 180 / 4286 (4.2%) | 340 | -1 | 1.1e-012 | 39 / 492 (7.9%) | 18 / 492 (3.7%) | 39 | -1 | 3.8e-003 | 14.0201 | | | | | | | |
| Motif | Logo | RC Logo | P-value | E-value | Sites | More | Submit/Download | Positional Distribution | Matches per Sequence |
| 32-GTTACCTRGCAA |  |  | 3.9e-003 | 2.0e-001 | 87 (1.8%) | ↧↥ | ⇢ |  |  |
| Details  | Train Positives | Train Positives | Train Negatives | Train DTC | Score | Test Positives | Test Positives | Test Negatives | Test DTC | P-value | Match Threshold | | --- | --- | --- | --- | --- | --- | --- | --- | --- | --- | --- | | 79 / 4266 (1.9%) | 2 / 4286 (0.0%) | 79 | -1 | 1.4e-021 | 8 / 492 (1.6%) | 0 / 492 (0.0%) | 8 | -1 | 3.9e-003 | 17.2474 | | | | | | | |
| Motif | Logo | RC Logo | P-value | E-value | Sites | More | Submit/Download | Positional Distribution | Matches per Sequence |
| 33-ACCAATCAG |  |  | 5.8e-003 | 3.0e-001 | 272 (5.7%) | ↧↥ | ⇢ |  |  |
| Details  | Train Positives | Train Positives | Train Negatives | Train DTC | Score | Test Positives | Test Positives | Test Negatives | Test DTC | P-value | Match Threshold | | --- | --- | --- | --- | --- | --- | --- | --- | --- | --- | --- | | 243 / 4266 (5.7%) | 83 / 4286 (1.9%) | 243 | -1 | 1.2e-019 | 29 / 492 (5.9%) | 12 / 492 (2.4%) | 29 | -1 | 5.8e-003 | 13.8256 | | | | | | | |
| Motif | Logo | RC Logo | P-value | E-value | Sites | More | Submit/Download | Positional Distribution | Matches per Sequence |
| 34-CGCCGCCGCC |  |  | 6.9e-003 | 3.6e-001 | 271 (5.7%) | ↧↥ | ⇢ |  |  |
| Details  | Train Positives | Train Positives | Train Negatives | Train DTC | Score | Test Positives | Test Positives | Test Negatives | Test DTC | P-value | Match Threshold | | --- | --- | --- | --- | --- | --- | --- | --- | --- | --- | --- | | 241 / 4266 (5.6%) | 99 / 4286 (2.3%) | 241 | -1 | 4.2e-015 | 30 / 492 (6.1%) | 13 / 492 (2.6%) | 30 | -1 | 6.9e-003 | 14.3497 | | | | | | | |
| Motif | Logo | RC Logo | P-value | E-value | Sites | More | Submit/Download | Positional Distribution | Matches per Sequence |
| 35-CAAGGTCAC |  |  | 1.0e-002 | 5.3e-001 | 505 (10.6%) | ↧↥ | ⇢ |  |  |
| Details  | Train Positives | Train Positives | Train Negatives | Train DTC | Score | Test Positives | Test Positives | Test Negatives | Test DTC | P-value | Match Threshold | | --- | --- | --- | --- | --- | --- | --- | --- | --- | --- | --- | | 461 / 4266 (10.8%) | 187 / 4286 (4.4%) | 461 | -1 | 6.3e-028 | 44 / 492 (8.9%) | 24 / 492 (4.9%) | 44 | -1 | 1.0e-002 | 12.9174 | | | | | | | |
| Motif | Logo | RC Logo | P-value | E-value | Sites | More | Submit/Download | Positional Distribution | Matches per Sequence |
| 36-CAAAGCCA |  |  | 1.4e-002 | 7.5e-001 | 1848 (38.8%) | ↧↥ | ⇢ |  |  |
| Details  | Train Positives | Train Positives | Train Negatives | Train DTC | Score | Test Positives | Test Positives | Test Negatives | Test DTC | P-value | Match Threshold | | --- | --- | --- | --- | --- | --- | --- | --- | --- | --- | --- | | 1660 / 4266 (38.9%) | 1223 / 4286 (28.5%) | 1660 | -1 | 2.1e-016 | 188 / 492 (38.2%) | 147 / 492 (29.9%) | 188 | -1 | 1.4e-002 | 9.05808 | | | | | | | |
| Motif | Logo | RC Logo | P-value | E-value | Sites | More | Submit/Download | Positional Distribution | Matches per Sequence |
| 37-CCACCACCWCS |  |  | 1.5e-002 | 7.6e-001 | 335 (7.0%) | ↧↥ | ⇢ |  |  |
| Details  | Train Positives | Train Positives | Train Negatives | Train DTC | Score | Test Positives | Test Positives | Test Negatives | Test DTC | P-value | Match Threshold | | --- | --- | --- | --- | --- | --- | --- | --- | --- | --- | --- | | 303 / 4266 (7.1%) | 102 / 4286 (2.4%) | 303 | -1 | 1.5e-024 | 32 / 492 (6.5%) | 16 / 492 (3.3%) | 32 | -1 | 1.5e-002 | 13.918 | | | | | | | |
| Motif | Logo | RC Logo | P-value | E-value | Sites | More | Submit/Download | Positional Distribution | Matches per Sequence |
| 38-VTGASTCAB |  |  | 1.6e-002 | 8.2e-001 | 615 (12.9%) | ↧↥ | ⇢ |  |  |
| Details  | Train Positives | Train Positives | Train Negatives | Train DTC | Score | Test Positives | Test Positives | Test Negatives | Test DTC | P-value | Match Threshold | | --- | --- | --- | --- | --- | --- | --- | --- | --- | --- | --- | | 551 / 4266 (12.9%) | 301 / 4286 (7.0%) | 551 | -1 | 4.3e-018 | 64 / 492 (13.0%) | 41 / 492 (8.3%) | 64 | -1 | 1.6e-002 | 12.0562 | | | | | | | |
| Motif | Logo | RC Logo | P-value | E-value | Sites | More | Submit/Download | Positional Distribution | Matches per Sequence |
| 39-CAGCAGCTGC |  |  | 1.8e-002 | 9.2e-001 | 582 (12.2%) | ↧↥ | ⇢ |  |  |
| Details  | Train Positives | Train Positives | Train Negatives | Train DTC | Score | Test Positives | Test Positives | Test Negatives | Test DTC | P-value | Match Threshold | | --- | --- | --- | --- | --- | --- | --- | --- | --- | --- | --- | | 521 / 4266 (12.2%) | 218 / 4286 (5.1%) | 521 | -1 | 9.0e-030 | 61 / 492 (12.4%) | 39 / 492 (7.9%) | 61 | -1 | 1.8e-002 | 12.3408 | | | | | | | |
| Motif | Logo | RC Logo | P-value | E-value | Sites | More | Submit/Download | Positional Distribution | Matches per Sequence |
| 40-AATGAATG |  |  | 2.0e-002 | 1.0e+000 | 284 (6.0%) | ↧↥ | ⇢ |  |  |
| Details  | Train Positives | Train Positives | Train Negatives | Train DTC | Score | Test Positives | Test Positives | Test Negatives | Test DTC | P-value | Match Threshold | | --- | --- | --- | --- | --- | --- | --- | --- | --- | --- | --- | | 253 / 4266 (5.9%) | 107 / 4286 (2.5%) | 253 | -1 | 4.6e-015 | 31 / 492 (6.3%) | 16 / 492 (3.3%) | 31 | -1 | 2.0e-002 | 14.7757 | | | | | | | |
| Motif | Logo | RC Logo | P-value | E-value | Sites | More | Submit/Download | Positional Distribution | Matches per Sequence |
| 41-CAAGATGGCG |  |  | 2.5e-002 | 1.3e+000 | 177 (3.7%) | ↧↥ | ⇢ |  |  |
| Details  | Train Positives | Train Positives | Train Negatives | Train DTC | Score | Test Positives | Test Positives | Test Negatives | Test DTC | P-value | Match Threshold | | --- | --- | --- | --- | --- | --- | --- | --- | --- | --- | --- | | 164 / 4266 (3.8%) | 40 / 4286 (0.9%) | 164 | -1 | 2.5e-019 | 13 / 492 (2.6%) | 4 / 492 (0.8%) | 13 | -1 | 2.5e-002 | 13.6636 | | | | | | | |
| Motif | Logo | RC Logo | P-value | E-value | Sites | More | Submit/Download | Positional Distribution | Matches per Sequence |
| 42-AAGAAAGAAA |  |  | 3.4e-002 | 1.8e+000 | 456 (9.6%) | ↧↥ | ⇢ |  |  |
| Details  | Train Positives | Train Positives | Train Negatives | Train DTC | Score | Test Positives | Test Positives | Test Negatives | Test DTC | P-value | Match Threshold | | --- | --- | --- | --- | --- | --- | --- | --- | --- | --- | --- | | 409 / 4266 (9.6%) | 142 / 4286 (3.3%) | 409 | -1 | 2.9e-031 | 47 / 492 (9.6%) | 30 / 492 (6.1%) | 47 | -1 | 3.4e-002 | 13.0258 | | | | | | | |
| Motif | Logo | RC Logo | P-value | E-value | Sites | More | Submit/Download | Positional Distribution | Matches per Sequence |
| 43-CCCAGCCC |  |  | 3.6e-002 | 1.8e+000 | 752 (15.8%) | ↧↥ | ⇢ |  |  |
| Details  | Train Positives | Train Positives | Train Negatives | Train DTC | Score | Test Positives | Test Positives | Test Negatives | Test DTC | P-value | Match Threshold | | --- | --- | --- | --- | --- | --- | --- | --- | --- | --- | --- | | 680 / 4266 (15.9%) | 452 / 4286 (10.5%) | 680 | -1 | 6.5e-012 | 72 / 492 (14.6%) | 51 / 492 (10.4%) | 72 | -1 | 3.6e-002 | 13.0078 | | | | | | | |
| Motif | Logo | RC Logo | P-value | E-value | Sites | More | Submit/Download | Positional Distribution | Matches per Sequence |
| 44-YCACGTGR |  |  | 3.6e-002 | 1.9e+000 | 651 (13.7%) | ↧↥ | ⇢ |  |  |
| Details  | Train Positives | Train Positives | Train Negatives | Train DTC | Score | Test Positives | Test Positives | Test Negatives | Test DTC | P-value | Match Threshold | | --- | --- | --- | --- | --- | --- | --- | --- | --- | --- | --- | | 585 / 4266 (13.7%) | 333 / 4286 (7.8%) | 585 | -1 | 3.9e-017 | 66 / 492 (13.4%) | 46 / 492 (9.3%) | 66 | -1 | 3.6e-002 | 11.7374 | | | | | | | |
| Motif | Logo | RC Logo | P-value | E-value | Sites | More | Submit/Download | Positional Distribution | Matches per Sequence |
| 45-CCCCACCC |  |  | 4.3e-002 | 2.2e+000 | 580 (12.2%) | ↧↥ | ⇢ |  |  |
| Details  | Train Positives | Train Positives | Train Negatives | Train DTC | Score | Test Positives | Test Positives | Test Negatives | Test DTC | P-value | Match Threshold | | --- | --- | --- | --- | --- | --- | --- | --- | --- | --- | --- | | 522 / 4266 (12.2%) | 353 / 4286 (8.2%) | 522 | -1 | 6.1e-009 | 58 / 492 (11.8%) | 40 / 492 (8.1%) | 58 | -1 | 4.3e-002 | 10.4536 | | | | | | | |
| Motif | Logo | RC Logo | P-value | E-value | Sites | More | Submit/Download | Positional Distribution | Matches per Sequence |
| 46-CATCTGTAA |  |  | 4.5e-002 | 2.3e+000 | 179 (3.8%) | ↧↥ | ⇢ |  |  |
| Details  | Train Positives | Train Positives | Train Negatives | Train DTC | Score | Test Positives | Test Positives | Test Negatives | Test DTC | P-value | Match Threshold | | --- | --- | --- | --- | --- | --- | --- | --- | --- | --- | --- | | 156 / 4266 (3.7%) | 51 / 4286 (1.2%) | 156 | -1 | 7.2e-014 | 23 / 492 (4.7%) | 12 / 492 (2.4%) | 23 | -1 | 4.5e-002 | 14.8619 | | | | | | | |
| Motif | Logo | RC Logo | P-value | E-value | Sites | More | Submit/Download | Positional Distribution | Matches per Sequence |
| 47-CCGCCTCC |  |  | 4.5e-002 | 2.4e+000 | 917 (19.3%) | ↧↥ | ⇢ |  |  |
| Details  | Train Positives | Train Positives | Train Negatives | Train DTC | Score | Test Positives | Test Positives | Test Negatives | Test DTC | P-value | Match Threshold | | --- | --- | --- | --- | --- | --- | --- | --- | --- | --- | --- | | 821 / 4266 (19.2%) | 595 / 4286 (13.9%) | 821 | -1 | 1.0e-009 | 96 / 492 (19.5%) | 73 / 492 (14.8%) | 96 | -1 | 4.5e-002 | 10.1336 | | | | | | | |
| Motif | Logo | RC Logo | P-value | E-value | Sites | More | Submit/Download | Positional Distribution | Matches per Sequence |
| 48-GACAGACA |  |  | 4.6e-002 | 2.4e+000 | 346 (7.3%) | ↧↥ | ⇢ |  |  |
| Details  | Train Positives | Train Positives | Train Negatives | Train DTC | Score | Test Positives | Test Positives | Test Negatives | Test DTC | P-value | Match Threshold | | --- | --- | --- | --- | --- | --- | --- | --- | --- | --- | --- | | 314 / 4266 (7.4%) | 181 / 4286 (4.2%) | 314 | -1 | 1.2e-009 | 32 / 492 (6.5%) | 19 / 492 (3.9%) | 32 | -1 | 4.6e-002 | 14.229 | | | | | | | |
| Motif | Logo | RC Logo | P-value | E-value | Sites | More | Submit/Download | Positional Distribution | Matches per Sequence |
| 49-GCGCAGGCGCA |  |  | 4.8e-002 | 2.5e+000 | 146 (3.1%) | ↧↥ | ⇢ |  |  |
| Details  | Train Positives | Train Positives | Train Negatives | Train DTC | Score | Test Positives | Test Positives | Test Negatives | Test DTC | P-value | Match Threshold | | --- | --- | --- | --- | --- | --- | --- | --- | --- | --- | --- | | 133 / 4266 (3.1%) | 35 / 4286 (0.8%) | 133 | -1 | 5.9e-015 | 13 / 492 (2.6%) | 5 / 492 (1.0%) | 13 | -1 | 4.8e-002 | 15.4248 | | | | | | | |
| Motif | Logo | RC Logo | P-value | E-value | Sites | More | Submit/Download | Positional Distribution | Matches per Sequence |
| 50-AACAACAACAA |  |  | 9.0e-002 | 4.7e+000 | 117 (2.5%) | ↧↥ | ⇢ |  |  |
| Details  | Train Positives | Train Positives | Train Negatives | Train DTC | Score | Test Positives | Test Positives | Test Negatives | Test DTC | P-value | Match Threshold | | --- | --- | --- | --- | --- | --- | --- | --- | --- | --- | --- | | 110 / 4266 (2.6%) | 13 / 4286 (0.3%) | 110 | -1 | 1.3e-020 | 7 / 492 (1.4%) | 2 / 492 (0.4%) | 7 | -1 | 9.0e-002 | 15.7514 | | | | | | | |
| Motif | Logo | RC Logo | P-value | E-value | Sites | More | Submit/Download | Positional Distribution | Matches per Sequence |
| 51-AGCCTGGGCTACA |  |  | 1.1e-001 | 5.9e+000 | 172 (3.6%) | ↧↥ | ⇢ |  |  |
| Details  | Train Positives | Train Positives | Train Negatives | Train DTC | Score | Test Positives | Test Positives | Test Negatives | Test DTC | P-value | Match Threshold | | --- | --- | --- | --- | --- | --- | --- | --- | --- | --- | --- | | 164 / 4266 (3.8%) | 17 / 4286 (0.4%) | 164 | -1 | 1.1e-031 | 8 / 492 (1.6%) | 3 / 492 (0.6%) | 8 | -1 | 1.1e-001 | 15.0511 | | | | | | | |
| Motif | Logo | RC Logo | P-value | E-value | Sites | More | Submit/Download | Positional Distribution | Matches per Sequence |
| 52-ACTGTAGCTGTCTTC |  |  | 1.9e-001 | 9.8e+000 | 85 (1.8%) | ↧↥ | ⇢ |  |  |
| Details  | Train Positives | Train Positives | Train Negatives | Train DTC | Score | Test Positives | Test Positives | Test Negatives | Test DTC | P-value | Match Threshold | | --- | --- | --- | --- | --- | --- | --- | --- | --- | --- | --- | | 81 / 4266 (1.9%) | 1 / 4286 (0.0%) | 81 | -1 | 1.7e-023 | 4 / 492 (0.8%) | 1 / 492 (0.2%) | 4 | -1 | 1.9e-001 | 16.0193 | | | | | | | |
| Stopped because 3 consecutive motifs exceeded the p-value threshold (0.05). | | | | | |
| --- | --- | --- | --- | --- | --- |
| STREME ran for 3043.51 seconds. | | | | | |

## Inputs & Settings

Previous Next Top

#### Sequences

| Role | Source | Alphabet | Sequence Count | Total Size |
| --- | --- | --- | --- | --- |
| Positive (primary) Sequences | neg50\_atac\_cleaned\_20211005.fasta | DNA | 4758 | 3999975 |
| Negative (control) Sequences | 2-Order Shuffled Positive Sequences | DNA | 4778 | 3999890 |

#### Background Model

**Source:** built from the negative (control) sequences

    **Order:** 2 (only order-0 shown)


| Name | Freq. | Bg. |  |  |  | Bg. | Freq. | Name |
| --- | --- | --- | --- | --- | --- | --- | --- | --- |
| Adenine | 0.229 | 0.229 | A | ~ | T | 0.229 | 0.229 | Thymine |
| Cytosine | 0.271 | 0.271 | C | ~ | G | 0.271 | 0.271 | Guanine |

#### Other Settings

|  |  |
| --- | --- |
| Strand Handling | This alphabet only has one strand. Only the given strand is processed. Both the given and reverse complement strands are processed. |
| Objective Function | Differential Enrichment |
| Statistical Test | Binomial Test |
| Motif Selection Criterion | Output motif with the lowest p-value on the training set each round. |
| Minimum Motif Width | 8 |
| Maximum Motif Width | 15 |
| Sequence Shuffling | Negative sequences are positives shuffled preserving 3-mer frequencies. |
| Test Set | 10% of the input sequences were randomly assigned to the test set. |
| Word Evaluation | Up to 25 words of each width from 8 to 15 were evaluated to find seeds. |
| Seed Refinement | Up to 4 seeds of each width from 8 to 15 were further refined. |
| Refinement Iterations | Up to 20 iterations were allowed when refining a seed. |
| Minimum Score | Match scoring was truncated if a match longer than 5 scored less than 0. |
| Refinement Match Subsets | A new motif was created from the optimal set of matches each refinement iteration. |
| Minimum Palindrome Ratio | 0.85 |
| Maximum Palindrome Edit Distance | 5 |
| Print Candidate Motifs? | No. |
| Random Number Seed | 0 |
| Total Length | The total length of each sequence set was limited to 4.00e+6. |
| Maximum Motif p-value | Stop when the p-value is greater than 0.05 for 3 consecutive motifs. |
| Maximum Motifs to Find | No maximum number of motifs. |
| Maximum Run Time | 14400 seconds. |

Previous Top

##### STREME version

5.4.1
(Release date: Sat Aug 21 19:23:23 2021 -0700)

##### Reference

Timothy L. Bailey,
"STREME: accurate and versatile sequence motif discovery",
*Bioinformatics*, Mar. 24, 2021.
[full text]

##### Command line

streme
--verbosity 1 --oc . --dna --totallength 4000000 --time 14400 --minw 8
--maxw 15 --thresh 0.05 --align center --dfile description --p
neg50\_atac\_cleaned\_20211005.fasta
